# Optimizing the light delivery of linear-array-based photoacoustic systems by double acoustic reflectors

**DOI:** 10.1038/s41598-018-31430-5

**Published:** 2018-08-29

**Authors:** Yuehang Wang, Rachel Su Ann Lim, Huijuan Zhang, Nikhila Nyayapathi, Kwang W. Oh, Jun Xia

**Affiliations:** 1 University at Buffalo, the State University of New York, Department of Biomedical Engineering, Buffalo, 14260 USA; 2 University at Buffalo, the State University of New York, Department of Electrical Engineering, Buffalo, 14260 USA

## Abstract

Although linear transducer arrays have been intensely used in photoacoustic imaging, their geometrical shape constrains light illumination. Today, most linear array based photoacoustic systems utilize side-illumination geometry, which consists of two line fiber bundles attached to the side of the probe. The angled light illumination increases the light travel distance in deep tissue, consequently limiting the imaging depth. This issue was partially addressed by adding a right angle prism in front of the transducer. While this design makes the light illumination and acoustic detection co-axial, the transducer and the fiber bundles are orthogonal to each other, making the system inconvenient for handheld use. To overcome this limitation, here we propose a double-reflector design, in which the second reflector redirects the acoustic signals by another 90°, so that the transducer and the fiber bundle are now parallel to each other. In this design, both the transducer and fiber bundle output are fitted into a compact housing for convenient handheld imaging. To evaluate the efficiency of our design, we performed various phantom and human *in vivo* experiments. Our results demonstrate that the double-reflector design indeed provides deeper imaging depth and it also allows for easy imaging of objects with uneven surfaces.

## Introduction

Photoacoustic (PA) imaging (PAI) is a promising modality for high-resolution imaging of optical absorption in deep tissue. PAI forms images based on the PA effect, in which absorbed light energies are converted into acoustic waves through thermal-elastic expansion. PAI takes advantage of the weak acoustic scattering in tissue to overcome the optical diffusion limitation. Thus, compared to conventional optical tomography techniques, such as diffuse optical tomography (DOT), PAI offers orders of magnitude better spatial resolution^1–4^.

**Figure 1:**
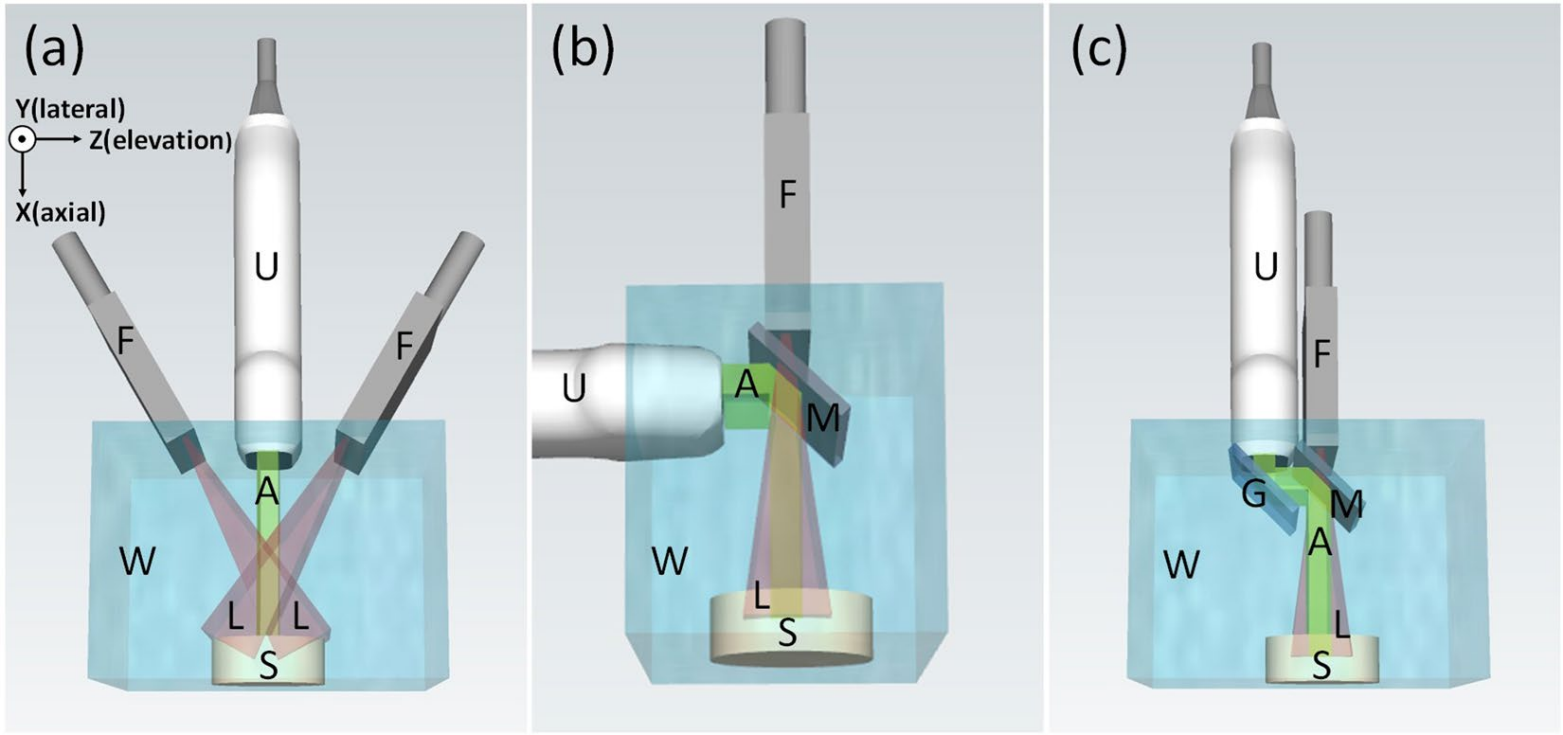
Schematic drawing of three different PA imaging systems. (**a**) Conventional side-illumination PAI system. (**b**) Single-reflector PAI system. (**c**) Double-reflector PAI system. U, ultrasound transducer array; F, fiber bundle; L, light beam; A, acoustic wave; W, water tank; S, sample; M, cold mirror; and G, glass. The laser beam is in red and the acoustic wave is in green.

Over the past few years, various PAI systems with different transducer arrays have been proposed. Among them, linear arrays are probably the most widely used transducers as they are affordable, convenient to use, and easily integrated with conventional ultrasound systems^5–10^. However, unlike hemispherical or annular array-based PAI systems, light illumination in a linear transducer array is quite challenging. Due to its geometric shape, light delivery can only be achieved from the side of the array as shown in Fig. 1a^11–14^. In this case, the light incident angle is critical for imaging performance. As we know, in biological tissue, photons will be absorbed and scattered multiple times before reaching the targeted imaging depth. Compared to that of perpendicular illumination, photons in the side-illumination geometry will need to travel over a longer distance before reaching the object, resulting in weaker fluence. Multiple groups have used the Monte Carlo (MC) simulation to investigate this issue^15,16^. All their results demonstrated that the smaller the light incident angle, the higher the light fluence in deep tissue.

Unfortunately, it is impossible to achieve a zero incident angle in the side-illumination system. Montilla *et al*. partially addressed this problem using a right angle prism^17^. As shown in Fig. 1b, this prism separates light illumination and signal detection into two directions. The prism is transparent to light, whereas the acoustic waves will be reflected by 90°, thus, achieving co-axially illumination and detection. However, this design is inconvenient for handheld operation because the transducer and fiber bundle are orthogonal to each other. Recently, Li *et al*. proposed a new design utilizing polymethyl methacrylate (PMMA) as an optical/acoustic coupler, which reflects the light beam two times and is permeable to acoustic waves^18^. This design achieves coaxial light delivery and acoustic detection with the optical fiber bundle and transducer probe parallel to each other. However, the acoustic impedance of PMMA and soft tissue are 3.33 and 1.58, respectively^19^, reducing the efficiency of acoustic propagation. In addition, because the sound speed of PMMA (2.484 mm/µs) is different from that of soft tissue (1.540 mm/µs), they utilized a fast marching method (FMM) based reconstruction algorithm^20^, which is more complex and time-consuming than standard back projection^21^.

To overcome the aforementioned limitations, we introduce a new co-axial illumination method based on a double-reflector concept as shown in Fig. 1c. Compared to the single-reflector design, we added one additional glass to reflect acoustic waves by another 90 degrees. Now, both the transducer array and fiber bundle are parallel to each other. The light illumination direction is also co-axial (co-planar) to the signal detection direction. Compared to the design by Li *et al*., we reflect acoustic waves instead of propagating them through two different mediums, therefore eliminating impedance mismatch.

## Results

### Imaging of tube phantoms

**Figure 2:**
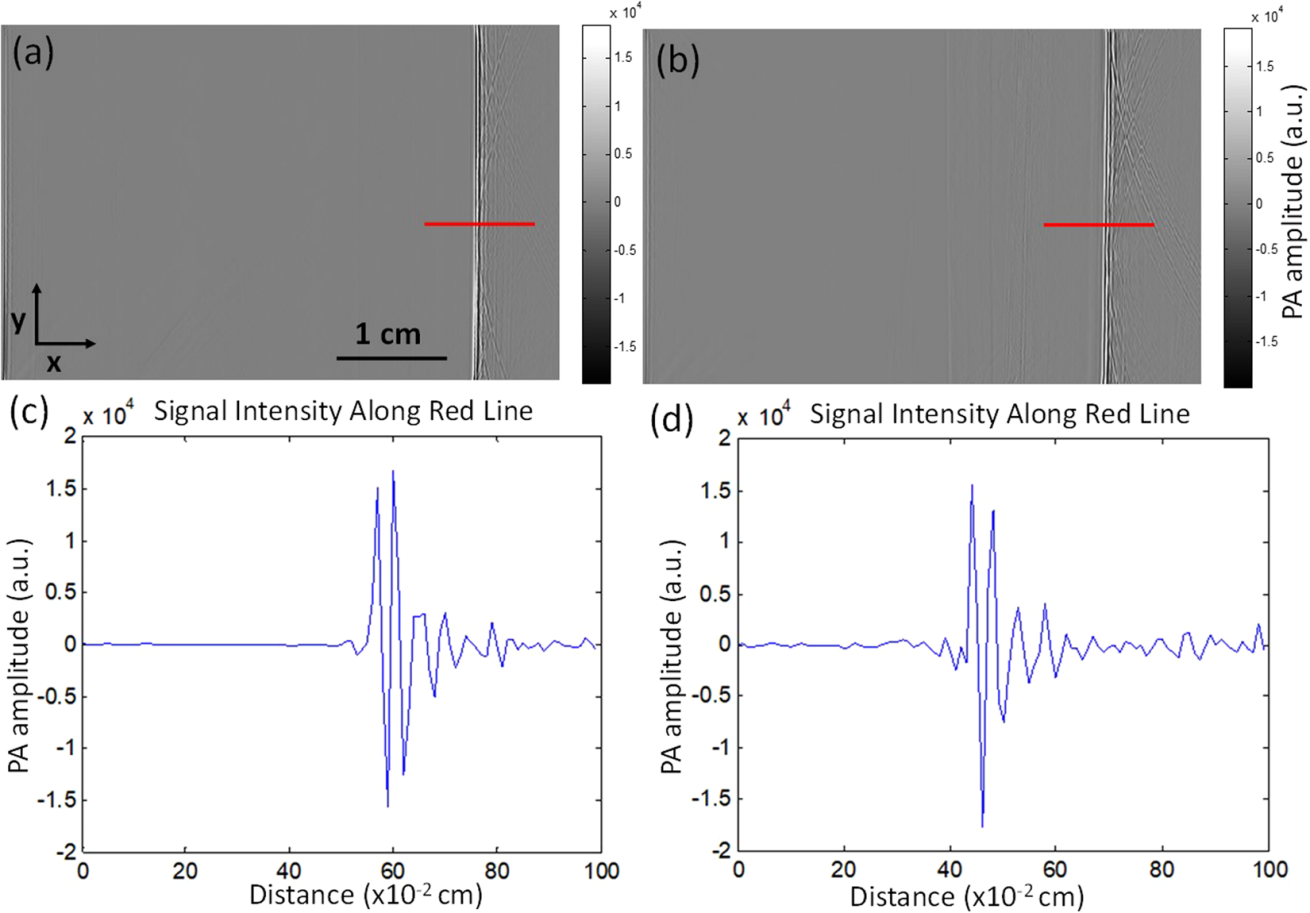
Raw PA data of single tube phantom experiment (**a**) Raw PA data acquire by the single-reflector system. (**b**) Raw PA data acquire by the double-reflector system. (**c**) Signal intensity along the red line in (**a)**. (**d**) Signal intensity along the red line in (**b**).

To investigate if the second reflector impacts the PA signal intensity, we first imaged a single tube filled with India ink, using both the single-reflector and double-reflector PAI systems. For both experiments, the transducer and tube distances were the same along the axial direction. The raw PA data are shown in Fig. 2. As shown in Fig. 2a,b, the position of the tube signals were both at approximately 5 cm, which means that the distance between the tube and the transducer surface were similar for both imaging systems. In addition, the color bars in Fig. 2a,b indicate that single and double-reflector systems have the same signal intensity range. For further analysis, we plot the signal intensity across the red solid lines in Fig. 2c,d. The signal profiles look very similar. The signal to noise ratios (SNR) are 39.2 and 38.6 for the single-reflector system and the double-reflector system, respectively and the peak-to-peak amplitudes are both around 3.25 (a.u.). These results demonstrated that the second reflector did not have much of an effect on the PA signal intensity.

**Figure 3:**
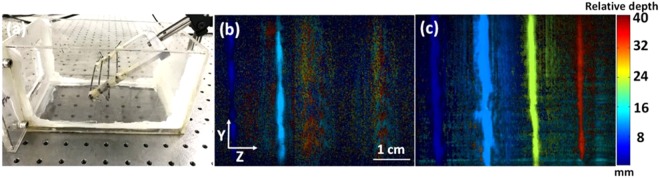
Four tubes phantom experiment. (**a**) Photo of four tubes phantom. (**b**) Depth-encoded MAP result of the side illumination method. (**c**) Depth-encoded MAP result of the double-reflector method. Double-reflector method exhibits a greater penetration depth and a more uniform light fluence.

To demonstrate that the double-reflector system has a slower light decay rate along the depth direction, we designed a phantom experiment using both side-illumination and double-reflector PAI systems. For this experiment, four tubes filled with pure India ink were placed at different depths with 1 cm of distance between each other. To mimic surrounding tissues, we used a mixture of 99.99% milk and 0.01% ink^22,23^. In order to cover the four tubes, we scanned over 5 cm in 50 s. Figure 3a shows the picture of the tube phantom, and Fig. 3b,c show the depth-encoded maximum amplitude projection (MAP) images. Here, different colors represent different depths. The double-reflector system clearly reveals all four tubes. Whereas, the side-illumination system is only able to show the two tubes closer to the light source. The maximum imaging depth for the double-reflector system is quantified to be 4 cm, while it is around 1.5 cm for the side-illumination system. These results demonstrate that, compared to the side-illumination system, the double-reflector system has a greater penetration depth and slower light fluence decay along the imaging depth.

### Imaging of a phantom with an uneven surface

**Figure 4:**
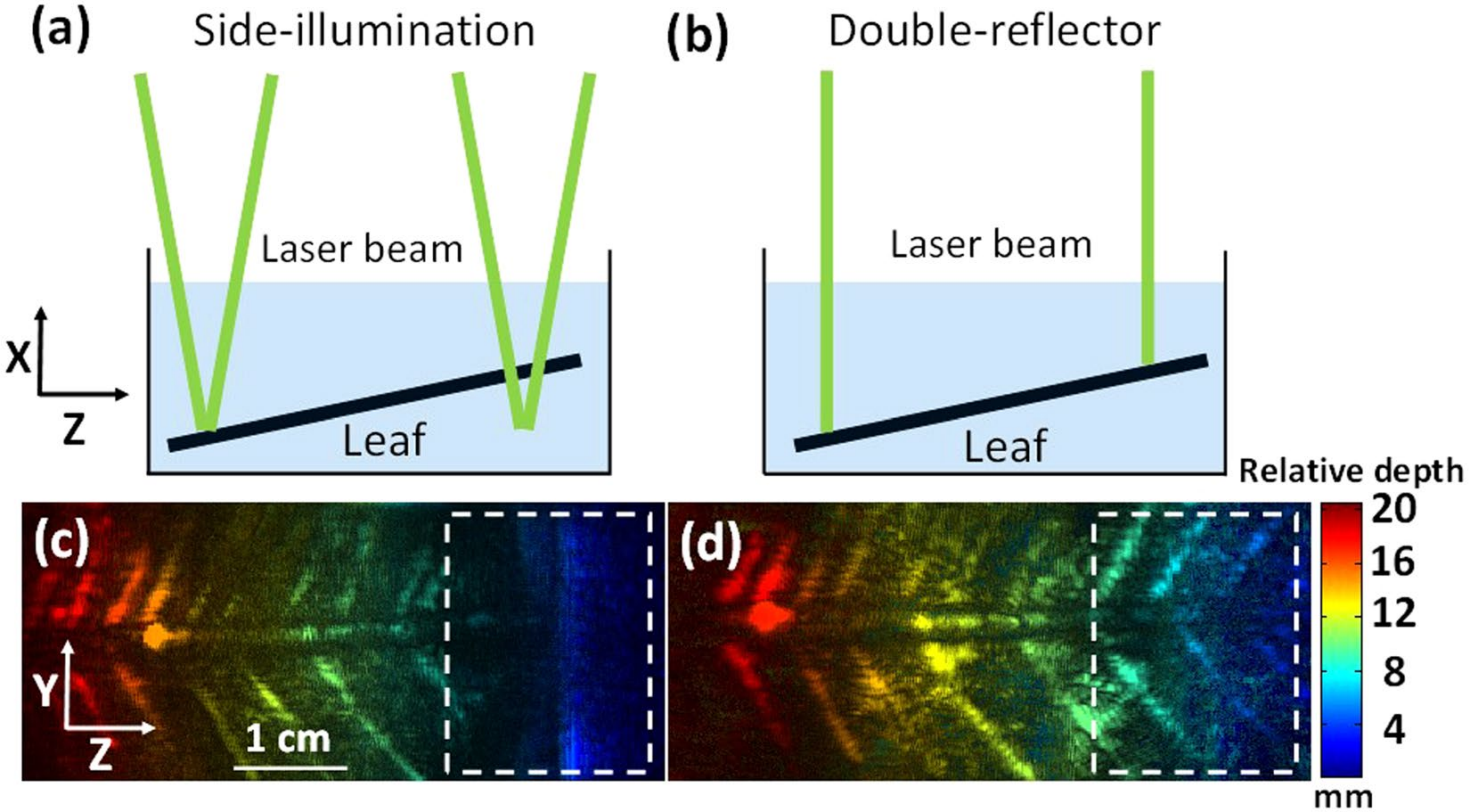
Depth-encoded MAP result of leaf phantom experiment. (**a**) Schematic of sideillumination geometry. (**b**) Schematic of double-reflector geometry. (**c**) Depth-encoded MAP image acquired by the side-illumination system. (**d**) Depth-encoded MAP image acquired by the double-reflector system. White dashed boxes indicate the shallow area of the leaf phantom.

The double-reflector method can also be utilized to image objects with uneven surfaces, especially when the object is immersed in coupling water or a bath of coupling gels, which are commonly used approaches in photoacoustic tomography^8,24^. To prove this capability, we imaged a tilted leaf skeleton in water. The laser setting was the same as the tube phantom experiment, and an area of 2.6 cm × 6 cm was imaged. Figure 4a and 4b show a schematic drawing of the laser beams. On the left side of Fig. 4a, two laser beams converge on the leaf surface, while on the right side of Fig. 4a, the two laser beams converge underneath the leaf, resulting in a dark field on the phantom surface. As for the double-reflector illumination geometry (Fig. 4b), one laser beam is delivered perpendicularly to the leaf phantom, so both the left and right sides of the leaf were well illuminated. Figure 4c and 4d show the leaf phantom results obtained by two illumination geometries, respectively. The depth-encoded color indicates the relative depth from the transducer to the leaf surface. As indicated by the white dashed box, there are no leaf features on the right side of Fig. 4c. The blue color indicates that the depth of this portion is shallow, which is where the laser beam could not be focused in the side-illumination case. In contrast, the perpendicular illumination in the double reflector set-up clearly reveals the entire leaf phantom. This result demonstrates that, compared to the side-illumination method, the double-reflector method offers a uniform light distribution for uneven surfaces.

### *In vivo* imaging of human forearm

**Figure 5:**
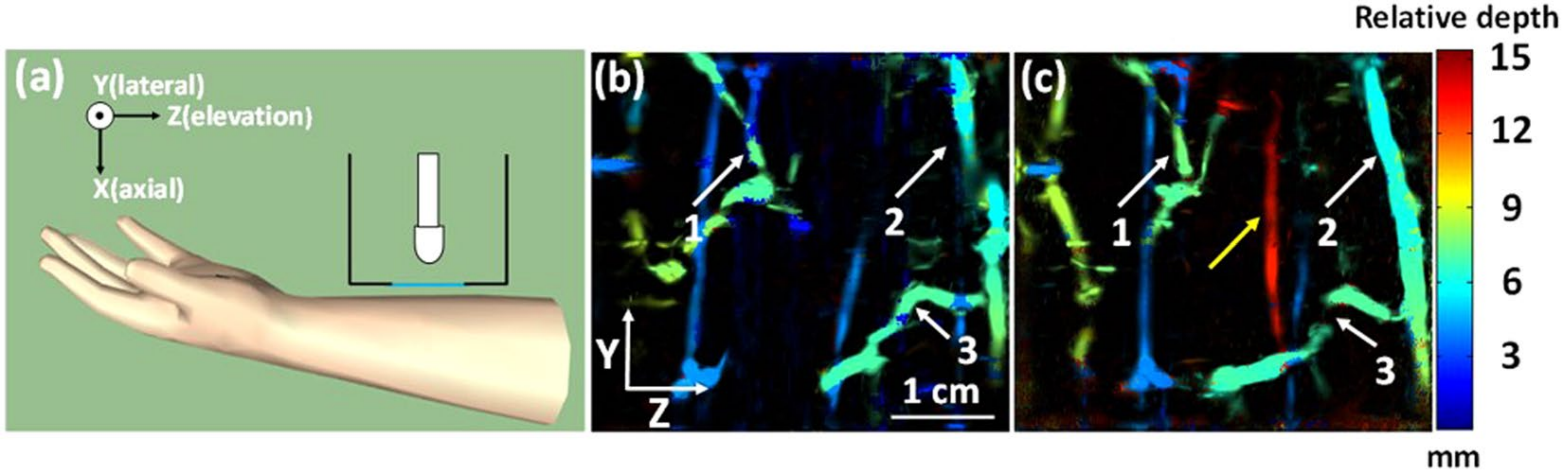
Human forearm experiment. (**a**) Schematic drawing of the forearm experiment setup. (**b**) Depth-encoded MAP image acquired by the side-illumination system. (**c**) Depth-encoded MAP image acquired by the doublereflector system. Arrows 1–3 point to the same features, while yellow arrow indicates the deepest vessel. Double-reflector illumination system has a deeper penetration depth of 15 mm.

We imaged the forearm of a volunteer to validate that our double-reflector system can image deeper vessels. For this experiment, an area of 3.4 cm × 3.8 cm was imaged. Figure 5a shows a schematic drawing of the experimental system. The blue solid line is the plastic film mentioned previously. The MAP images acquired by the side-illumination and the double-reflector systems are shown in Fig. 5b,c, respectively. The depth encoded color indicates the relative depth of vessels from the skin surface. Similar vascular features in both images are identified by three white arrows (arrows 1–3). In terms of penetration depth, as shown in the color bar, red color corresponds to the deepest vessel, while blue indicates shallow features. In Fig. 5c, a red vessel at around 15 mm deep can be seen as indicated by a yellow arrow. However, there are no red vessels in the forearm image acquired by the side-illumination system, and the deepest vessel is around 9 mm. This result further proves the advantage of double-reflection illumination.

## Discussion

In summary, we have developed a double-reflector illumination method for linear-array-based PAI systems. This design allows for co-axial light delivery and acoustic detection and the entire setup is very compact for handheld use. We demonstrated the performance of our system in both phantom and human studies. Compared to existing PAI systems based on the side-illumination geometry, the double-reflector illumination geometry provides a greater imaging depth. Also, the perpendicular light delivery mechanism provides uniform illumination for objects with uneven surfaces. Compared to the single-reflector system, our double-reflector design makes the ultrasound probe and the optical fiber bundles to be parallel to each other. With the 3D printed transducer and fiber bundle holder, the whole mount is very compact and can be easily adapted in any linear transducer arrays. In future studies, linear arrays with a large lateral field of view can be adapted to our system, which will be good for imaging large objects such as the human breast. For free-hand operation, we may also utilize a 3D-printed mount to enclose the transducer array and the coupling water. The whole probe can then be handheld and scanned as in a conventional ultrasound. With broad availability of linear arrays, we expect our method to further advance the image quality and applications in PAI.

## Methods

### System configuration

**Figure 6:**
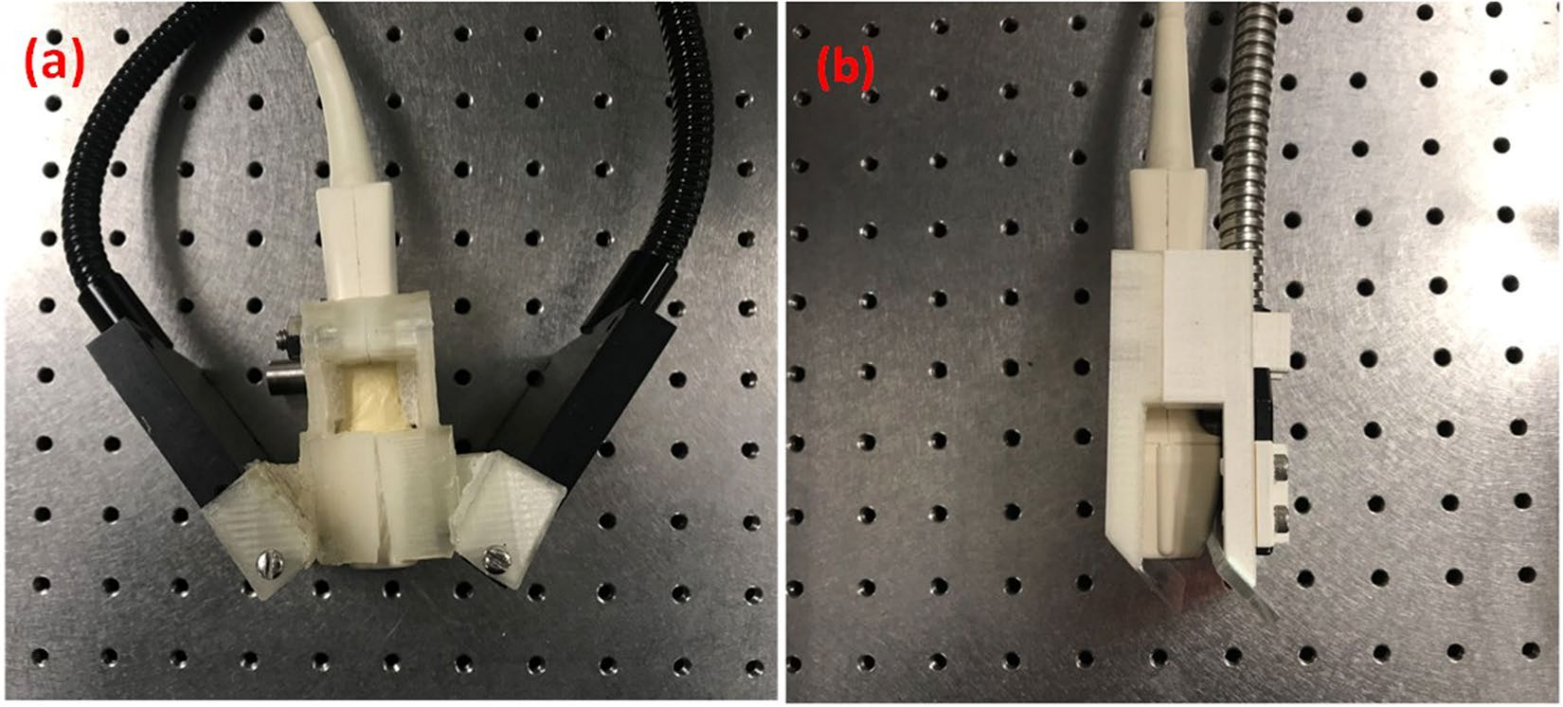
Photographs of integrated linear array and fiber bundle of side and double-reflector illumination geometries. (**a**) Integrated linear array and fiber bundle of side-illumination PAI system. (**b**) Integrated linear array and fiber bundle of double-reflector illumination PAI system. All mounts were 3D printed.

The PAI system used in this study consists of a 10-ns-pulsed Nd:YAG laser with 10 Hz pulse repetition rate and 1064 nm output wavelength, an ATL L7-4 transducer array with 5 MHz central frequency and 128 elements, and a Verasonics’ Vantage data acquisition system with 128 receive channels. The Vantage system allows us to adjust the time gain compensation (TGC) at different depths so that the strong skin signals caused by bright-field illuminations can be attenuated. The maximum amplification of the system is 54 dB and the sampling rate is 20 MHz (four times the central frequency of the array). The Nd:YAG laser was produced by Continuum Lasers and the pulse energy is around 700 mJ. For the side-illumination setup, as shown in Fig. 6a, we used a bifurcated fiber bundle with a 1.1-cm-diameter circular input and two 7.5-cm-length line outputs (Light CAM #2, Schott Fostec). These two fiber bundles were tilted to focus at around 25 mm under the transducer surface (the acoustic focus of the transducer). During the experiment, the fiber bundles were mounted on a lab-made holder and were moved simultaneously with the transducer (Fig. 6a). For both the single-reflector setup and the double-reflector setup, the fiber bundles we used has a 0.8-cm-diameter circular input and 5-cm-length line output (Moritex Corporation). For all the three setups, the fiber bundles received the same input laser energy (~700 mJ). While the light intensity on the subject surface might be different in the side-illumination and double-reflector cases (due to the different number of fiber output lines). We did not change the laser power to compensation that difference, because we would like to investigate what is the best illumination scheme for a given laser output power.

To avoid optical reflection on the acoustic reflector, we used a high-performance TECHSPEC® cold mirror (Edmund Optics Inc.). The mirror is 2.5 cm × 5 cm in size, featuring a multi-layer dielectric coating optimized for greater than 97% transmission at 1064 nm wavelength under 45° incident angle. The mirror is nontransparent to acoustic waves and reflects the incoming photoacoustic waves by 90°. The second reflector used in the double-reflector setup is a conventional glass with the same dimensions as the first reflector. The two reflectors are parallel to each other. Since the total elements size of the linear array is 3.8 cm × 1.5 cm, the size of both the cold mirror and the glass are large enough to cover the entire transducer array surface. To combine all the components, we also designed a 3D printed mount as shown in Fig. 6b. It can be seen that the double-reflector mount is more compact and convenient to use than the side-illumination mount (Fig. 6a).

### Phantom study

For all phantom experiments, we immersed the objects into a lab-made water tank. The scanning direction is along the elevation direction of the transducer, with a scanning step size of 0.1 mm and speed of 1 mm/s. The phantom is stationary, while the transducer and optical fiber bundle were mounted on a translation stage and moved together. The translation stage was driven by a NEMA 23 motor.

### *In vivo* study

As for *in vivo* study, a cuboid water tank with an opening in the bottom was used. The opening was sealed with a plastic film and the forearm was coupled to the film with ultrasound gel. The transducer was submerged in the water tank and scanned horizontally (along the elevation direction of the transducer array). The scanning step size and the speed were the same as the phantom study.

All human imaging studies have been reviewed and approved by the University at Buffalo Institutional Review Board (IRB). All experiments were performed in accordance with relevant guidelines and regulations. In addition, we have obtained informed consents from all subjects.

### Laser safety

For imaging with different setups, the input laser energy was the same and the surface intensity was all below the 100 mJ/cm^2^ limit defined by ANSI^25^.

### Image reconstruction

Since the double-reflector geometry increases the distance between the object and transducer, the elevation spatial resolution will be poorer at deeper depths^1^. To address this issue, we used the focal-line-based 3D image reconstruction algorithm, which improves the elevation resolution of all depths up to the width of the acoustic focus^26^. To reduce computation time, 3D reconstruction was performed throuhg a NVIDIA Titan X Pascal GPU. For better visualization, all reconstructed 3D images were projected along the axial direction of the transducer array to form a depth encoded MAP image. All computations were performed in Matlab.

## Data Availability

The datasets generated and/or analyzed during the current study are available from the corresponding author upon reasonable request.
